# Exosomal miR‐181d‐5p Derived from Rapamycin‐Conditioned MDSC Alleviated Allograft Rejection by Targeting KLF6

**DOI:** 10.1002/advs.202304922

**Published:** 2023-10-23

**Authors:** Chao Wei, Yaru Sun, Fanxing Zeng, Xiunian Chen, Li Ma, Xiaoxue Liu, Xiaolin Qi, Weiyun Shi, Hua Gao

**Affiliations:** ^1^ State Key Laboratory Cultivation Base Shandong Provincial Key Laboratory of Ophthalmology Eye Institute of Shandong First Medical University Qingdao 266071 China; ^2^ Eye Hospital of Shandong First Medical University (Shandong Eye Hospital) Jinan 250117 China; ^3^ School of Ophthalmology Shandong First Medical University & Shandong Academy of Medical Science Jinan 250117 China

**Keywords:** allograft rejection, exosome, KLF6, miR‐181d‐5p, myeloid‐derived suppressor cells

## Abstract

Immune rejection and side effects of long‐term administration of immunosuppressants are the two major obstacles to allograft acceptance and tolerance. The immunosuppressive extracellular vesicles (EVs)‐based approach has been proven to be effective in treating autoimmune/inflammatory disorders. Herein, the anti‐rejection advantage of exosomes (Rapa‐Exo) from rapamycin‐conditioned myeloid‐derived suppressor cells (MDSCs) over exosomes (Exo‐Nor) from the untreated MDSCs is shown. The exosomal small RNA sequencing and loss‐of‐function assays reveal that the anti‐rejection effect of Rapa‐Exo functionally relies on miR‐181d‐5p. Through target prediction and double‐luciferase reporter assay, Kruppel‐like factor (KLF) 6 is identified as a direct target of miR‐181d‐5p. Finally, KLF6 knockdown markedly resolves inflammation and prolongs the survival of corneal allografts. Taken together, these findings support that Rapa‐Exo executes an anti‐rejection effect, highlighting the immunosuppressive EVs‐based treatment as a promising approach in organ transplantation.

## Graphical abstract

This study has revealed that the exosome released from rapamycin‐conditioned MDSCs (Rapa‐Exo) significantly prolongs the survival of corneal allografts. Mechanistically, the identified exosomal miR‐181d‐5p directly targets KLF6, which then results in the lowered expression of proinflammatory cytokines, eventually contributing to the resolution of inflammation and the great improvement of corneal allograft rejection.

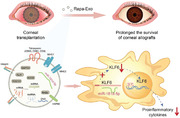

## 1 Introduction

Transplantation is the major therapy for end‐stage organ diseases, and immune rejection is the primary reason for graft failure.^[^ ^1^, ^2^, ^3^ ^]^ Although organ transplantation has advanced markedly, wide and long‐term application of immunosuppressants increases the risk and susceptibility to various complications, such as neurotoxicity, hyperlipidemia, and infectious and neoplastic diseases.^[^ ^4^, ^5^, ^6^ ^]^ Moreover, immunosuppressive drugs (such as calcineurin inhibitors) can disrupt the graft‐protective immune response.^[^ ^7^, ^8^, ^9^ ^]^ To circumvent these dilemmas, there is an urgent need to find novel mechanisms and targets to induce immune tolerance and treat allograft rejection in the absence of long‐term immunosuppression.^[^ ^10^ ^]^

Extracellular vesicles (EVs), with nano‐scale phospholipid bilayer structure, are secreted by living cells and play crucial roles in major (patho) physiological processes, including intercellular communications, transmission of signaling molecules, metabolism regulation, and cancer development.^[^ ^11^, ^12^, ^13^, ^14^ ^]^ Emerging studies reveal that EVs have several superiorities over conventional drug carriers, characterized by high biocompatibility, prominent structural stability, high loading capacity, and low immunogenicity, endowing great potential as a novel drug delivery system.^[^ ^15^, ^16^, ^17^, ^18^, ^19^, ^20^ ^]^ Accumulating evidence demonstrated the availability and feasibility of EVs‐based strategy for disease treatment, including cancer,^[^ ^21^, ^22^ ^]^ myocardial infarction,^[^ ^23^, ^24^ ^]^ spinal cord injury,^[^ ^25^ ^]^ cartilage defects,^[^ ^26^, ^27^ ^]^ and wound healing.^[^ ^28^, ^29^ ^]^

Based on the immunomodulatory properties of the immunosuppressive cells, the immunosuppression can also be critically initiated by their EVs, such as regulatory T cells,^[^ ^30^, ^31^ ^]^ myeloid‐derived‐suppressor‐cells (MDSCs),^[^ ^32^, ^33^ ^]^ and mesenchymal stem cells.^[^ ^34^, ^35^ ^]^ Notably, the immunosuppressive EVs‐based approach is effective in treating various inflammatory/autoimmune diseases, including infectious diseases,^[^ ^36^, ^37^, ^38^ ^]^ rheumatoid arthritis,^[^ ^39^, ^40^ ^]^ multiple sclerosis,^[^ ^41^, ^42^ ^]^ and allograft rejection.^[^ ^43^, ^44^, ^45^, ^46^ ^]^ However, the application and mechanisms underlying exosome‐based therapy in allograft rejection have not been fully addressed.

Using the murine corneal transplantation model, we revealed an anti‐rejection effect of rapamycin (Rapa) nano‐micelle,^[^ ^47^, ^48^ ^]^ mechanistically associated with the enhancement of MDSCs^’^ immunosuppressive function.^[^ ^47^ ^]^ Some studies highlighted the potential applications of MDSC‐derived EVs in preclinical autoimmune/inflammatory disease models, especially in rheumatoid arthritis,^[^ ^49^, ^50^ ^]^ inflammatory bowel disease,^[^ ^51^ ^]^ and autoimmune alopecia areata.^[^ ^52^ ^]^ The proteomic approach also showed the enriched molecular cargo with immunosuppressive functions in MDSC exosomes.^[^ ^53^, ^54^ ^]^ However, the effect and mechanisms underlying the MDSC‐released exosomes in allogeneic immune response remain largely uncertain, including exosomes derived from Rapa‐preconditioned MDSCs (Rapa‐Exo).

In the present study, the murine corneal transplantation model was employed to evaluate the critical role of MDSC‐released exosomes in allograft rejection. In addition to its pathogenesis similar to other organ transplantation rejection, the murine corneal transplantation model has advantages in investigating immune rejection,^[^ ^3^ ^]^ including continuous and real‐time observation of alloimmune response and neovascularization, without host sacrifice. Our results showed that the anti‐rejection effect of Rapa‐Exo was superior to the exosomes derived from the untreated MDSCs, and associated with exosomal miR‐181d‐5p. Mechanistically, miR‐181d‐5p directly targeted Kruppel‐like factor (KLF) 6 to resolve inflammation and subsequently prolong the survival of corneal allografts. Collectively, these findings indicated that exosomal miR‐181d‐5p from Rapa‐treated MDSCs alleviates transplant rejection by directly targeting KLF6, providing a new avenue for treatment of allograft rejection.

## 2 Results and Discussion

### 2.1 Characterization of Rapa‐Exo

**Figure 1:**
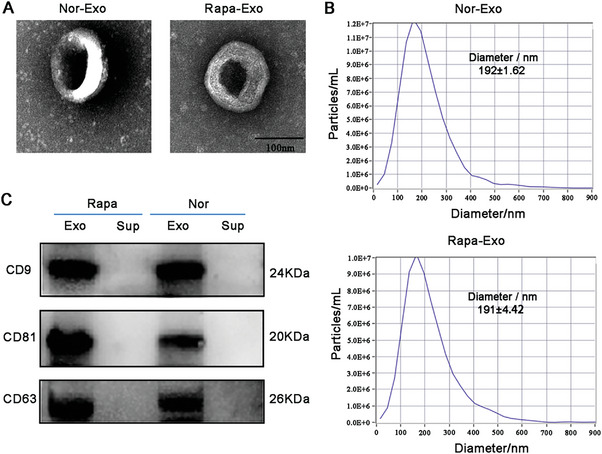
Characterization of Rapa‐Exo with different treatments. A) Representative TEM images of exosomes from MDSCs in the presence or absence of Rapa. Scale bar, 100 nm. B) Size distributions of exosomes derived from MDSCs in the presence or absence of Rapa determined using NAT. C) WB analysis of EV markers in different groups. Exo, exosome; Sup, MDSC supernatant.

To characterize Rapa‐Exo, the EVs were isolated by ultracentrifugation, using standard procedures. As shown in **Figure** **1**A, the transmission electron microscope (TEM) images revealed that the exosomes obtained from MDSCs in the presence or absence of Rapa displayed a representative exosome morphology with a round or cup‐shaped structure. The size of exosomes from different origins was evaluated using nanoparticle tracking analysis (NTA) (Figure 1B). Those isolated from MDSCs with or without Rapa treatment showed a bell‐shaped curve, with an average exosome size of 192±1.62 nm (Nor‐Exo) and 191±4.42 nm (Rapa‐Exo). Moreover, the significant expression of surface markers (CD9, CD81, and CD63) in exosomes isolated from Rapa‐treated MDSCs was observed through Western blot (WB), similar to exosomes obtained from untreated MDSCs (Figure 1C).

### 2.2 Rapa‐Exo Significantly Alleviated Allograft Rejection in a Well‐Characterized Murine Corneal Transplantation Model

**Figure 2:**
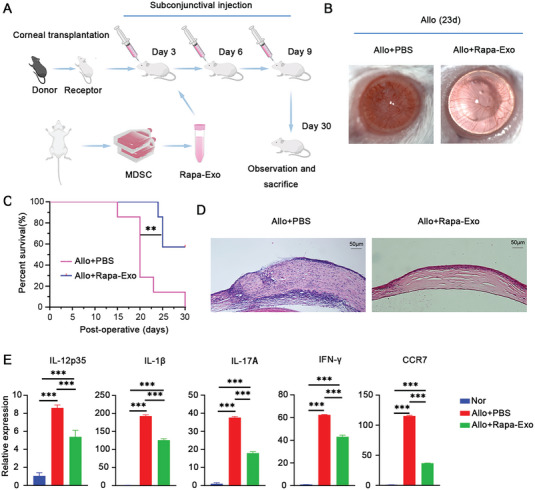
Rapa‐Exo alleviated corneal allograft rejection. A) The schematic procedure of strategy for topically treating corneal allograft rejection by Rapa‐Exo. B) The representative slit‐lamp images of corneal grafts after treatment with Rapa‐Exo on postoperative day 23 (*n* = 3 per group). C) The survival curve of corneal allografts topically treated with Rapa‐Exo, analyzed by Kaplan–Meier method (*n* = 6–8 per group). D) Histopathological alterations of corneal allografts with different managements by H&E staining (*n* = 3 per group). Scale bar 50 µm. E) Quantitative analysis of the pro‐inflammatory cytokines (IL‐12p35, IL‐1β, IL‐17A , and IFN‐γ) and CCR7 in Rapa‐Exo‐treated corneal grafts via real‐time PCR (*n* = 3 per group, in pools of three samples). ^**^ *p* < 0.01, ^***^ *p* < 0.001.

Accumulating evidence reveals the great application prospect of immunosuppressive EV‐based therapeutic strategies for treating inflammatory diseases and inhibiting allograft rejection.^[^ ^38^, ^46^, ^55^, ^56^, ^57^, ^58^ ^]^ Moreover, our previous findings underscored the excellent anti‐rejection effect of Rapa nano‐micelle ophthalmic solution through MDSCs.^[^ ^47^, ^48^ ^]^ Thus, we speculated that Rapa‐Exo delayed the allograft rejection better than exosomes derived from untreated MDSCs (Nor‐Exo). To test this assumption, the anti‐rejection effect of exosomes with different origins was investigated in a well‐established murine corneal transplantation model (**Figure** **2**A). As shown in Figure S1A–C (Supporting Information), compared with the receipt treated with phosphated‐buffered saline (PBS) (Allo+PBS) or Nor‐Exo (Allo+Nor‐Exo), subconjunctive injection of Rapa‐Exo substantially delayed corneal transplantation rejection, manifested by corneal grafts with lightened edema and opacity, prolonged survival time, and less inflammatory infiltrations. These findings indicated that Rapa‐Exo had an anti‐rejection advantage over Exo from untreated MDSCs.

Subsequently, we mainly evaluated the effect and outcomes of Rapa‐Exo on allograft rejection. The corneal allografts treated with PBS showed shortened survival time, with serious edema and opacity, as well as more corneal neovascularization (Figure 2B,C). Interestingly, after three conjunctival injections of Rapa‐Exo, the survival of corneal allografts was significantly extended, accompanied by transparent but less neo‐vascularized corneas (Figure 2B,C). Histopathological examination showed that rejected corneal samples presented disorganized tissue structure and more inflammatory infiltration (Figure 2D), and highly‐expressed pro‐inflammatory cytokines (including IL‐12p35, IL‐1β, IL‐17A, and IFNγ) and C‐C chemokine receptor 7 (CCR7) (Figure 2E). In contrast, the corneal allografts after topic administration of Rapa‐Exo showed a well‐organized structure and reduced infiltration of inflammatory cells (Figure 2D), as well as lowered expression of pro‐inflammatory cytokines and CCR7 (Figure 2E), which suggested a prominent improvement of corneal allograft rejection. Additionally, we also observed the distribution of Rapa‐Exo labeled with red fluorescence in corneal allografts and anterior chamber using immunofluorescence staining (Figure S2, Supporting Information). Overall, these results demonstrated that Rapa‐Exo with strong immunomodulatory properties, remarkably alleviated allograft rejection, superior to Nor‐Exo.

### 2.3 The Anti‐Rejection Effect of Rapa‐Exo Relied on miR‐181d‐5p

**Figure 3:**
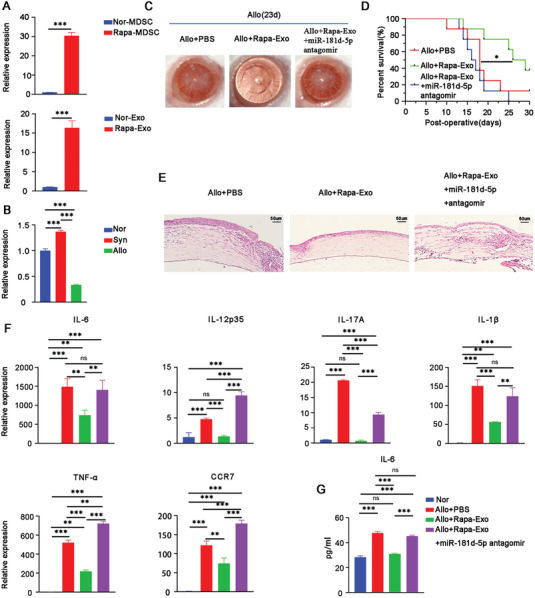
The anti‐rejection outcome of Rapa‐Exo was closely related to miR‐181d‐5p. A) Quantitative analysis of miR‐181d‐5p expression in Rapa‐Exo and Rapa‐treated MDSCs by real‐time PCR, respectively. B) The transcriptional level of miR‐181d‐5p in corneal grafts with different groups. Nor, Normal group; Syn, synergic group; Allo, Allogeneic group (*n* = 3 per group, in pools of three samples). C) The typical slit‐lamp images of corneal allografts with different treatments (*n* = 3 per group). Allo+PBS, the allogeneic group with the subconjunctival treatment of PBS; Allo+Rapa‐Exo, the allogeneic group topically administrated with Rapa‐Exo; Allo+Rapa‐Exo+miR‐181d‐5p antagomir, the allogeneic group topically administrated with Rapa‐Exo in the presence of miR‐181d‐5p antagomir. D) The survival of corneal allografts with different managements analyzed via Kaplan–Meier approach (*n* = 6–8 per group). E) The pathological alterations of corneal allografts with various conditions were evaluated using H&E staining. Scale bar 50 µm. F) The mRNA levels of pro‐inflammatory cytokines (IL‐6, IL‐12p35, IL‐17A, IL‐1β, and TNF‐α) and CCR7 in allografts determined using real‐time PCR (n = 3/group, in pools of 3 samples). G) The protein levels of IL‐6 in corneal grafts with different conditions as quantified by enzyme‐linked immunosorbent assay (ELISA). ^*^ *p* < 0.05, ^**^ *p* < 0.01, ^***^ *p* < 0.001, n.s, no significance.

Next, we conducted exosomal small RNA sequencing for Rapa‐Exo and Nor‐Exo to elucidate the mechanism by which Rapa‐Exo delays corneal allograft rejection. As shown in the volcano plot, 42 differentially expressed miRNAs were identified in Rapa‐Exo (12 down‐and 30 upregulated miRNAs) (Figure S3A, Supporting Information). Gene Ontology (GO) analysis revealed that the target genes of differentially expressed miRNAs were functionally associated with various biological processes, including immune system process and innate immune response (Figure S3B, Supporting Information). The heatmap exhibited 20 differentially expressed miRNAs involved in immunomodulation (Figure S3C, Supporting Information), miR‐181d‐5p was selected for subsequent investigation because of its high expression after Rapa treatment and functional relevance to inflammation resolution and immunomodulatory property^[^ ^59^, ^60^, ^61^ ^]^ (Figure S3A,C, Supporting Information). Furthermore, the increased miR‐181d‐5p in Rapa‐Exo compared to Nor‐Exo was confirmed using real‐time PCR and in Rapa‐treated MDSCs (**Figure** **3**A). On the other hand, miR‐181d‐5p expression decreased in rejected corneal allografts (Allo) as compared with Nor and Syn corneal samples (Figure 3B). These results suggested the possible involvement of miR‐181d‐5p in Rapa‐Exos’ anti‐rejection effect.

In order to further determine whether miR‐181d‐5p plays a role in the anti‐rejection effect of Rapa‐Exo, loss‐of‐function experiments were performed in murine corneal transplantation models. The antisense oligonucleotides of mouse miR‐181d‐5p (miR‐181d‐5p antagomir) and Rap‐Exo were subconjunctively injected into the recipients, and then corneal allograft rejection was assessed. As shown in Figure 3C,D, compared to the recipient mouse treated with PBS, topical application of Rapa‐Exo significantly delayed immune rejection, presented transparent cornea with less neovascularization, and prolonged the survival time. Surprisingly, after co‐treatment with miR‐181d‐5p antagomir, the anti‐rejection effect of Rapa‐Exo was impaired, showing aggravated immune rejection, along with serious edema and opacity, corneal neovascularization, and shortened survival time (Figure 3C,D). Hematoxylin‐eosin (H&E) staining displayed nearly normal corneal structure and reduced infiltrations of inflammatory cells in Rapa‐Exo‐treated corneal allografts, but disordered corneal structure with more inflammatory infiltrations after co‐treatment with miR‐181d‐5p antagomir (Figure 3E). In addition, the levels of pro‐inflammatory cytokines (interleukin (IL)−6, IL‐12p35, IL‐1β, IL‐17A, and tumor necrosis factor‐alpha (TNF‐α)) and CCR7 in Rapa‐Exo‐treated corneal allografts were much lower than in PBS‐treated grafts (Figure 3F,G). Nonetheless, the reduced expression of pro‐inflammatory cytokines and CCR7 in Rapa‐treated corneal allografts was reversed after co‐treatment with miR‐181d‐5p antagomir (Figure 3F,G). Taken together, these findings indicated that miR‐181d‐5p inhibition abolishes the anti‐rejection effect of Rapa‐Exo.

### 2.4 Exosomal miR‐181d‐5p Resolved Lipopolysaccharides (LPS)‐Induced Inflammatory Response In Vitro

**Figure 4:**
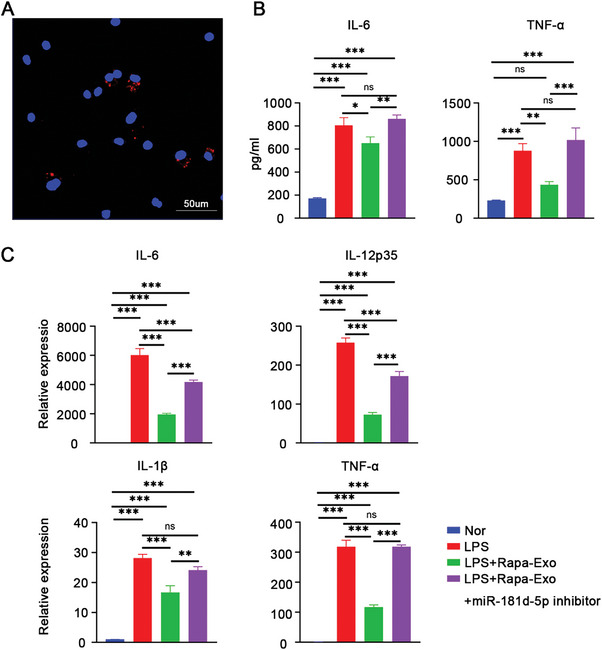
Exosomal miR‐181d‐5p significantly inhibited inflammatory response in LPS‐challenged BMDMs. A) The confocal image of BMDMs after incubation with EvLINK 555‐labeled‐Rapa‐EV (100 µg mL^−1^) for 24 h. Scale bar 50 µm. B) The concentrations of IL‐6 and TNF‐α in the supernatants of BMDMs under different conditions quantified by ELISA. C) The mRNA levels of pro‐inflammatory cytokines (IL‐6, IL‐12p35, IL‐1β, and TNF‐α) in BMDMs with different treatments by real‐time PCR. Nor, the BMDMs without any treatment; LPS, the BMDMs stimulated with LPS (100 ng mL^−1^); LPS+Rapa‐Exo, the BMDMs co‐incubated with LPS (100 ng mL^−1^) and Rapa‐Exo (100 µg mL^−1^); LPS+Rapa‐Exo+ miR‐181d‐5p inhibitor, the BMDMs co‐treated with LPS (100 ng mL^−1^) and Rapa‐Exo (100 µg mL^−1^) in presence of miR‐181d‐5p inhibitor (100 µg mL^−1^). ^*^ *p* < 0.05, ^**^ *p* < 0.01, ^***^ *p* < 0.001, n.s, no significance.

To better explore the immunomodulatory role of exosomal miR‐181d‐5p, the LPS‐stimulated bone marrow‐derived macrophage (BMDM) model was established. As shown in **Figure** **4**A, the immunofluorescence signaling of Rapa‐Exo labeled with red fluorescence (EvLINK 555) was observed in BMDMs, suggesting an effective uptake of exosomes by BMDMs. Compared to LPS‐challenged BMDMs, the levels of pro‐inflammatory cytokines including IL‐6, IL‐12p35, IL‐1β, and TNF‐α in LPS‐stimulated BMDMs were dramatically lowered after co‐incubation with Rapa‐Exo (Figure 4B,C). However, when co‐treated with miR‐181d‐5p inhibitor, the anti‐inflammatory property of Rapa‐Exo was impaired, as manifested by sharp elevation of pro‐inflammatory cytokines (Figure 4B,C). To address the direct immunomodulatory effect of miR‐181d‐5p on LPS‐induced inflammatory response, miR‐181d‐5p mimic and miR‐181d‐5p mimic NC were used to transfect BMDMs, respectively. As expected, the expression of pro‐inflammatory cytokines in miR‐181d‐5p mimic/LPS‐treated BMDMs was much lower than that in LPS or LPS/miR‐181d‐5p mimic NC‐treated BMDMs (Figure S4A,B, Supporting Information), which was consistent with the findings obtained in LPS‐ stimulated BMDMs co‐treated with Rapa‐Exo and miR‐181d‐5p inhibitor. These results demonstrated that exosomal miR‐181d‐5p inhibits the inflammatory response in vitro.

### 2.5 miR‐181d‐5p Directly Targeted KLF6

**Figure 5:**
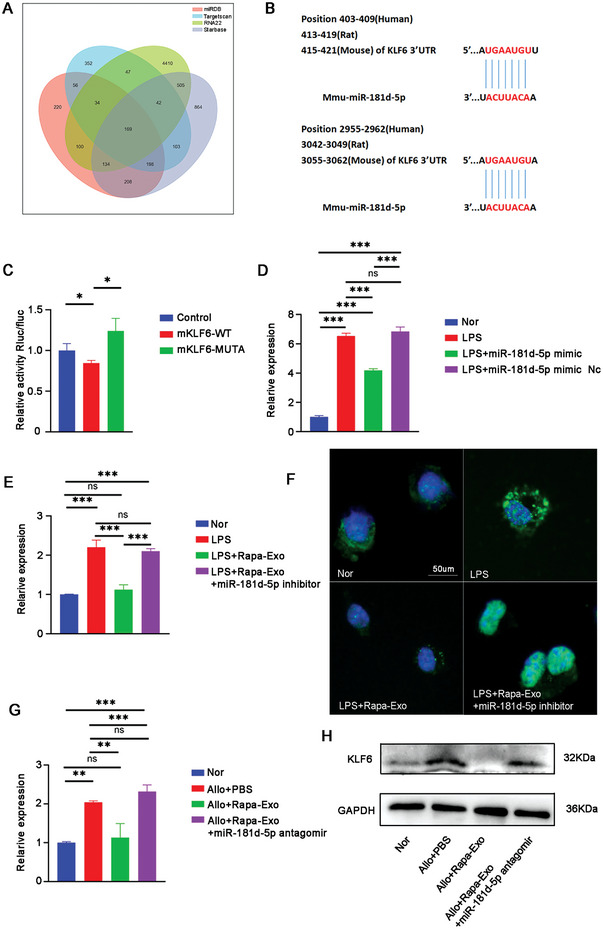
KLF6 was the downstream target of miR‐181d‐5p. A) Venn diagram displaying the predicted targets of miR‐181d‐5p through four different databases. B) The illustration of predicted 3′‐UTR binding sites for KLF6 of miR‐181d‐5p. C) The binding potential of miR‐181d‐5p with KLF6 examined using dual‐luciferase activity assay. D) The transcriptional level of KLF6 in BMDMs with different conditions determined using real‐time PCR. Nor, the untreated BMDMs; LPS, the BMDMs challenged with LPS (100 ng mL^−1^); LPS+miR‐181d‐5p mimic, the BMDMs co‐treated with LPS (100 ng mL^−1^) and miR‐181d‐5p mimic (100 µg mL^−1^); LPS+miR‐181d‐5p mimic NC, the BMDMs stimulated with LPS (100 ng mL^−1^) in the presence of miR‐181d‐5p mimic NC (100 µg mL^−1^). E) The KLF6 mRNA in BMDMs with different treatments quantified by real‐time PCR, as in (cf. 4B). F) The expression and location of KLF6 in LPS‐challenged BMDMs in the presence or absence of Rapa‐Exo and miR‐181d‐5p inhibitor, as in (cf. 4B). Scale bar 50 µm. G) The mRNA levels of KLF6 in corneal allografts evaluated through real‐time PCR (*n* = 3 per group, in pools of three samples). Allo+PBS, the allogeneic mice with the subconjunctival treatment of PBS; Allo+Rapa‐Exo, the allogeneic mice topically treated with Rapa‐Exo; Allo+Rapa‐Exo+miR‐181d‐5p antagomir, the allogeneic group topically administrated with Rapa‐Exo in the presence of miR‐181d‐5p antagomir. H) Immunoblotting analysis of KLF6 in corneal grafts with different treatments (*n* = 3 per group, in pools of three samples) (as in cf. 5G). ^*^ *p* < 0.05, ^**^ *p* < 0.01, ^***^ *p* < 0.001, n.s, no significance.

Next, we determined the target of miR‐181d‐5p to inhibit the inflammatory response. Using four miRNA target prediction platforms (miRDB, Targetscan, RNA22, and Starbase), 169 targets of miR‐181d‐5p were obtained (**Figure** **5**A). After comparison and screening, KLF6 was the selected candidate for further investigation based on its critical role in modulating inflammatory response,^[^ ^62^, ^63^, ^64^, ^65^ ^]^ the potential binding sites of 3′‐untranslated region (3′‐UTR) of KLF6 with miR‐181d‐5p are shown in Figure 5B. Double‐luciferase reporter assay revealed that the luciferase activity was significantly inhibited by binding miR‐181d‐5p with the 3′‐UTR of KLF6 reporter (Figure 5C). However, the inhibition of luciferase activity was impaired in the case of a mutation in the binding site of KLF6 reporter (Figure 5C). These results were supported by the findings of Zhang et al.^[^ ^66^ ^]^

In LPS‐challenged BMDMs, the transcriptional level of KLF6 in LPS/ miR‐181d‐5p mimic‐treated BMDM was much lower than in LPS‐stimulated BMDMs, but without significant reduction in LPS/ miR‐181d‐5p mimic NC treated BMDMs (Figure 5D). Furthermore, the expression of KLF6 in LPS‐challenged BMDMs was also dramatically downregulated when incubated with Rapa‐Exo. This inhibitory role of Rapa‐Exo was counteracted when BMDMs were transfected with miR‐181d‐5p inhibitor (Figure 5E). Immunofluorescence staining analysis showed lowered expression and reduced nuclear translocation of KLF6 in LPS‐challenged BMDMs after incubation with Rapa‐Exo, which was restored after transfection with miR‐181d‐5p inhibitor (Figure 5F). Importantly, the expression of KLF6 in corneal allografts was also decreased after treatment with Rapa‐Exo, but reversed when co‐treated with Rapa‐Exo and miR‐181d‐5p antagomir (Figure 5G,H). In summary, these findings demonstrated that KLF6 is a direct target for miR‐181d‐5p in inflammation regulation and allograft rejection.

### 2.6 Knockdown of *KLF6* Remarkably Extinguished the Inflammatory Response and Alleviated Allograft Rejection

**Figure 6:**
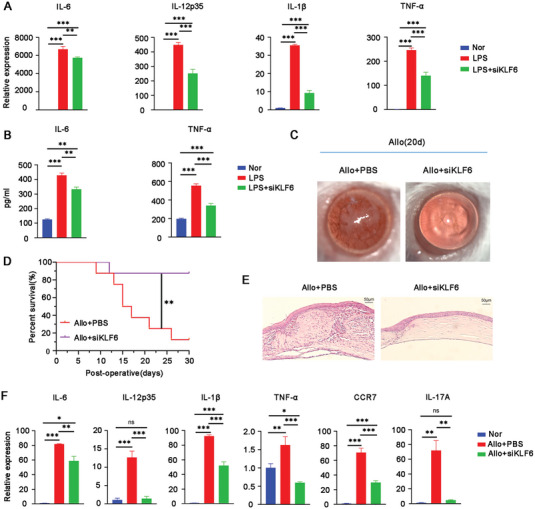
Knockdown of KLF6 significantly blocked inflammatory response and delayed corneal immune rejection. A) The expression of pro‐inflammatory cytokines in LPS (100 ng mL^−1^) ‐stimulated BMDMs in the presence or absence of siKLF6 (100 µg mL^−1^) for 24 h, respectively. B) The protein levels of IL‐6 and TNF‐α in supernatants of LPS‐stimulated BMDMs in the presence or absence of siKLF6 for 24 h. C) The representative slit‐lamp photographs of corneal allografts treated with or without siKLF6 on postoperative day 20 (*n* = 3 per group). D) The survival of corneal allografts with different treatments evaluated by Kaplan–Meier approach (*n* = 6–8 per group). E) The pathological alterations of corneal allografts on postoperative day 20 examined using H&E staining (*n* = 3 per group). Scale bar 50 µm. F) The transcriptional levels of pro‐inflammatory cytokines (IL‐6, IL‐12p35, IL‐1β, TNF‐α, and IL‐17A) and CCR7 in allografts were quantified by real‐time PCR (*n* = 3 per group, in pools of three samples). Allo+PBS, the allogeneic group with subconjunctival treatment of PBS; Allo+siKLF6, the allogeneic group topically administrated with siKLF6 (100 µg mL^−1^). ^*^ *p* < 0.05, ^**^ *p* < 0.01, ^***^ *p* < 0.001, n.s, no significance.

Finally, we examined the effect of KLF6 on LPS‐induced inflammatory response and corneal allograft rejection. As shown in Figure S5A,B (Supporting Information), the expression of KLF6 in LPS‐stimulated BMDMs after transfection of siKLF6 was much lower than in LPS‐stimulated BMDMs without transfection, indicating a successful knockdown of *KLF*6. Compared to LPS‐incubated BMDMs without transfection, the expression of pro‐inflammatory cytokines including IL‐6, IL‐12p35, IL‐1β, and TNF‐α was decreased in LPS‐incubated BMDMs after transfection of siKLF6 (**Figure** **6**A,B). These results indicated that the reduced KLF6 was closely associated with the resolved inflammatory responses in vitro.

Subsequently, we evaluated the outcome of KLF6 knockdown on corneal allograft rejection. Based on slit‐lamp assay, we observed that the topical intervention with siKLF6 significantly delayed corneal allograft rejection than in the un‐transfected corneal grafts, characterized by transparent corneas, less corneal neovascularization, and prolonged survival time (Figure 6C,D). These findings were similar to those obtained from Rapa‐Exo treatment. Histopathological analysis revealed that the PBS‐treated corneal grafts had an elevated inflammatory infiltration with disordered structure, while transfected grafts showed reduced inflammatory infiltration and well‐organized tissue structure (Figure 6E). Additionally, similar to the rejected grafts, the allografts transfected with siKLF6 showed decreased expression of pro‐inflammatory cytokines, such as IL‐6, IL‐12p35, IL‐1β, IL‐17A, and TNF‐α, as well as CCR‐7 (Figure 6F). Together, these results demonstrated that targeting KLF6 by siRNA significantly inhibits the inflammatory response and prolongs the survival of corneal allografts.

Although the immunomodulatory role of miR‐181d‐5p and KLF6 has been documented in several physiological and pathological conditions, but their associations with allograft rejection remain unknown. In this study, for the first time, we addressed the anti‐rejection effect of the exosomal miR‐181d‐5p/KLF6 axis using a corneal transplantation model. Nonetheless, the present has several limitations. First, the mechanism underlying rapamycin‐elevated expression of miR‐181d‐5p needs to be explored. Second, although the inhibitory effect of miR‐181d‐5p on KLF6 has been determined, the binding efficiency and interaction relationships of the two miR‐181d‐5p binding sites in 3′‐UTR of KLF6 should be investigated further based on the predicted potential binding sites. Third, despite the better anti‐rejection effect of Rapa‐Exo than Nor‐Exo, a comparison with the existing anti‐rejection drugs or means is required for subsequent clinical applications. Finally, the effectiveness‐validation of Rapa‐Exo in other organ transplantation models should also be investigated.

## 3 Conclusion

**Figure 7:**
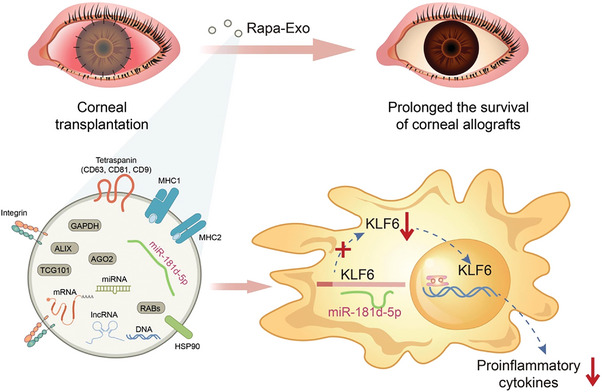
Illustration of Rapa‐Exo alleviated allograft rejection directly through miR‐181d‐5p/KLF6 signaling. The miR‐181d‐5p in Rapa‐Exo resolved the inflammatory response by directly targeting KLF6, through which Rapa‐Exo inhibited the inflammation and ameliorated corneal allograft rejection.

Our findings revealed that Rapa‐Exo showed a better anti‐rejection effect than Nor‐Exo. The anti‐rejection effect of Rapa‐Exo closely relied on miR‐181d‐5p. Mechanistically, exosomal miR‐181d‐5p inhibited pro‐inflammatory response by targeting KLF6, ultimately alleviating the allograft rejection (**Figure** **7**). This study highlighted Rapa‐Exo as an effective immunosuppressive EVs for treating immune rejection and identified miR‐151d‐5p /KLF6 as a potential therapeutic signaling pathway.

## 4 Experimental Section

### Animals

Six to eight weeks old C57BL/6 or BALB/c male mice used in this study were purchased from Beijing Vital River Laboratory Animal Technology Company Limited. All experiments were carried out in accordance with the Committee guidelines of Shandong Eye Institute ([2021] No.53) and the Association for Research in Vision and Ophthalmology (ARVO) Statement for the Use of Animals in Ophthalmic and Vision Research.

### MDSCs Isolation and Treatment

The MDSCs from the bone marrow of BALB/c male mice were purified using a myeloid‐derived suppressor cell isolation kit (130‐094‐538, Miltenyi Biotec) according to the manufacturer's protocol. Then the isolated MDSCs were incubated with 10% exosome‐free fetal bovine serums (Gibco) containing rapamycin (100 nmol mL^−1^, MedChemExpress) for 2 days. After treatment, the supernatants and MDSCs were collected for further applications.

### Exosome Preparations, Identification, and Labeling

Exosomes were isolated through ultracentrifugation, as described previously.^[^ ^67^ ^]^ Briefly, the supernatant was collected by centrifugation at 1000 g for 10 min and clarified to eliminate cell debris and macro‐particles, at 2000 g for an additional 20 min. The supernatants were pooled by ultra‐filtration through a 100 kDa molecular weight cut‐off membrane (Thermo Fisher Scientific) at 10 000 g for 30 min. The concentrated supernatant was subjected to ultracentrifugation at 1 10 000 g for 60 min. After re‐suspension and ultracentrifugation, the pellet was suspended in PBS for subsequent application. TEM, NTA, and WB were applied to identify the exosomes. The exosomes were labeled with red fluorescence (EvLINK 555, illuTINGO) and injected subconjunctivally to trace the distribution in ocular tissues.

### Exosomal Small RNA Sequencing and Bioinformatic Analysis

Total RNA from Rapa‐Exo and Nor‐Exo was isolated using mirVana miRNA Isolation Kit (Ambion) according to the manufacturer's protocol, respectively. After quantification and integrity assessment of total RNA, the TruSeq Small RNA Sample Pre Kits (Illumina) was used to construct a small RNA library. The quality was evaluated and the library was sequenced by OE Biotech Co., Ltd. (Shanghai, China) using DNA High Sensitivity Chips and Illumina HiSeq X Ten platform, 150 bp paired‐end reads were generated.

The clean raw data were acquired through base calling and quality control treatment. Clean reads were then obtained by filtration of low‐quality reads, 5′ primer contaminants, and poly (A), followed by length screening and removal of the reads without 3′adapter and insert tag. For analysis, the clean raw data were used to detect the known and novel miRNAs. The known miRNAs were identified using miRBase v22 database (http://www.mirbase.org/), and the novel miRNAs were analyzed by mirdeep2. The differentially expressed miRNAs were calculated and filtered with the threshold of |log2 (FC)| ≥ 1 and *p*‐value < 0.05. Miranda Software Miranda was applied to predict the targets of differentially expressed miRNAs. GO enrichment analysis of differentially expressed miRNA‐target‐gene was performed using GOseq R packages based on the hypergeometric distribution. The RNA sequencing dataset was deposited in the OMIX, China National Center for Bioinformation/Beijing Institute of Genomics, Chinese Academy of Sciences (accession no.CRA011734).

### Murine Corneal Transplantation and Treatment

Corneal transplantation was established as described previously.^[^ ^68^ ^]^ Briefly, the corneal grafts with 2 mm from C57BL/6 mice were fixed in BALB/C mice's central graft beds with 1.5 mm in diameter. Ofloxacin eye ointment was applied to prevent infection, and cornea grafts with severe complications were excluded from the experiments. Slit‐lamp microscopy was used to examine the corneal allografts, and immune rejection was determined based on corneal opacity, edema, and neovascularization according to the scoring system.^[^ ^69^ ^]^

In order to evaluate the effect of Rapa‐Exo on corneal allograft rejection, the recipient mice were subconjunctivally injected with 10 µL Rapa‐Exo (100 µg mL^−1^) on postoperative days 3, 6, and 9, while PBS and Nor‐Exo were used as controls. To determine the correlation between Rapa‐Exo and miR‐181d‐5p on corneal transplant rejection, the recipient mice received 10 µL mixture containing Rapa‐Exo (100 µg mL^−1^) and miR‐181d‐5p antagomir (1000 µg mL^−1^, GenePharma, Shanghai, China) through subconjunctival injection on postoperative days 3, 6, and 9. PBS and Rapa‐Exo were used as controls. Finally, the effect of KLF6 on corneal allograft rejection was assessed, and the recipient mice were subconjunctivally transfected with siKLF6 (1000 µg mL^−1^, GenePharma, Shanghai, China) on postoperative days 3, 6, and 9, and PBS was used as the control. The sequences of miR‐181d‐5p antagomir and siKLF6 are listed in (Tabel S1, Supporting Information).

### BMDMs Culture, LPS Challenge, and Treatment

BMDMs were induced using M‐CSF (50 ng mL^−1^, Proteintech, Wuhan, China) according to a previous protocol.^[^ ^69^ ^]^ To investigate the effect of Rapa‐Exo and miR‐181d‐5p on LPS‐stimulated inflammation response, miR‐181d‐5p mimic (1 µg), miR‐181d‐5p inhibitor (1 µg), and control miRNA(1 µg) were transfected using lipofectamine 3000 reagents (Invitrogen) and OPTI‐MEM (Invitrogen) at room temperature for 15 min, respectively. Subsequently, the BMDMs were challenged using LPS in the presence or absence of Rapa‐Exo (100 µg). The supernatant and cells were harvested for subsequent investigations. miR‐181d‐5p mimic, miR‐181d‐5p inhibitor, and control miRNA were obtained from GenePharma (Shanghai), and the sequences are listed in (Tabel S1, Supporting Information). Moreover, the exosomes were also labeled with EvLINK 555 to determine their location with BMDMs.

### Real‐Time PCR

Total RNA was extracted from corneal samples, exosomes, or cells using TRIzol reagent (12183555, Invitrogen), and the concentration was determined on a NanoDrop One spectrophotometer (Thermo Fisher). Reverse transcription was carried out using HiScript III RT SuperMix (Vazyme). Then, quantitative real‐time PCR was carried out on an ABI prism 7500 (Applied Biosystems) with miRNA Universal SYBR qPCR Master Mix (Vazyme). GAPDH and U6 were adopted to normalize the data, and the relative expression of genes was determined using the comparative threshold method (2^−△△Ct^). The primers used are listed in (Tabel S2, Supporting Information).

### WB

The total proteins of exosomes, cellular samples, or corneal tissues were extracted using RIPA lysis buffer containing protease inhibitors and quantified using Bradford assay. The total proteins were separated by SDS‐PAGE and transferred to a PVDF membrane (Millipore). Then, the membranes were probed with primary antibodies at 4 °C overnight and then incubated with the donkey anti‐rabbit or anti‐mouse second antibody conjugated with horseradish peroxidase (Cell Signaling Technology) to detect the target proteins using an Immobilon Western Chemiluminescent HRP Substrate kit (WBKLS0500, Millipore). The antibodies utilized are as follows: anti‐CD9 (ab223052, Abcam), anti‐CD81 (ab109201, Abcam), anti‐CD63 (ab217345, Abcam), anti‐KLF6 (14716‐1‐AP, Proteintech, Chicago, IL, USA), and anti‐GAPDH (5174, Cell Signaling Technology, Danvers, MA, USA).

### Enzyme‐Linked Immunosorbent Assay (ELISA)

According to the manufacturer's protocols, the protein levels of IL‐6 and TNFα in the supernatant of LPS‐challenged BMDMs and corneal samples were determined using commercial mouse ELISA kits (KE00085 and KE00080, Proteintech).

### H&E Staining and Immunofluorescence Staining

After washing with cold PBS and fixing with 4% paraformaldehyde, the challenged BMDMs were permeabilized using 0.25% Triton X‐100 for 10 min before incubation with rabbit anti‐KLF6 antibody (14716‐1‐AP, Proteintech). Alexa Fluor 488‐conjugated donkey anti‐rabbit secondary antibody (A21206, Invitrogen) was used to label the primary antibody, and DAPI was applied for counterstaining. The images were acquired using Zeiss LSM 880 laser‐scanning confocal microscope and ZEN 2010 software (Carl Zeiss).

The murine eyeballs with different groups were collected and fixed with tissue fixative (Citotest, Nantong, China) for 24 h. After dehydration and paraffin embedding, 4 µm thick sections were sliced and stained in a Ventana Discovery XT Immunstainer (Roche Diagnostics, Basel, Switzerland). The images were obtained under a microscope (Olympus, Tokyo, Japan).

### Luciferase Reporter Assays

Luciferase activity assays were conducted as described previously.^[^ ^70^ ^]^ The wild‐type KLF6 3′UTR firefly luciferase reporter plasmids and KLF6 3′UTR firefly luciferase reporter plasmids with the potential miR‐181d‐5p binding site mutation were co‐transfected with miR‐181d‐5p mimics or miR‐NC mimics into human embryonic kidney 293 cells, respectively, while Renilla luciferase reporter plasmids were transfected as internal controls. The luciferase activities were measured using a Dual‐Glo Luciferase Assay Kit (Promega) 48 h post‐transfections.

### Statistical Analysis

All experiments were performed in triplicate, and the data are presented as mean ± standard deviation (SD). Two‐tailed Student's *t*‐test was used for the comparison between two groups, and one‐way analysis of variance (ANOVA) with Tukey's post‐hoc comparison test was applied for more than two groups. The survival of allografts was analyzed using the Kaplan–Meier method. *p* < 0.05 was considered statistically significant.

## Conflict of Interest

The authors declare no conflict of interest.

## Supporting information

Supporting Information

## Data Availability

The data that support the findings of this study are available from the corresponding author upon reasonable request.
