# Supplementary material for: Exosomal miR‐181d‐5p Derived from Rapamycin‐Conditioned MDSC Alleviated Allograft Rejection by Targeting KLF6
Source: Adv Sci (Weinh). 2023 Oct 23;10(34):2304922. doi: 10.1002/advs.202304922 (PMC10700181; doi:10.1002/advs.202304922)
Supplement: Supplementary file 1 — Supporting Information [file ADVS-10-2304922-s001.pdf]

ADVANCED SCIENCE
Open Access

Supporting Information

for *Adv. Sci.*, DOI 10.1002/advs.202304922

Exosomal miR-181d-5p Derived from Rapamycin-Conditioned MDSC Alleviated Allograft Rejection by Targeting KLF6

*Chao Wei, Yaru Sun, Fanxing Zeng, Xiunian Chen, Li Ma, Xiaoxue Liu, Xiaolin Qi, Weiyun Shi and Hua Gao**

Supporting Information

Exosomal miR-181d-5p Derived from Rapamycin-Conditioned MDSC Alleviated Allograft Rejection by Targeting KLF6

*Chao Wei, Yaru Sun, Fanxing Zeng, Xiunian Chen, Li Ma, Xiaoxue Liu, Xiaolin Qi, Weiyun Shi, Hua Gao**

C. Wei, Y. Sun, F. Zeng, X. Chen, L. Ma, X. Liu

State Key Laboratory Cultivation Base, Shandong Provincial Key Laboratory of Ophthalmology, Eye Institute of Shandong First Medical University, Qingdao266071, China

X. Qi, W. Shi, H. Gao

State Key Laboratory Cultivation Base, Shandong Provincial Key Laboratory of Ophthalmology, Eye Institute of Shandong First Medical University, Qingdao266071, China

Eye Hospital of Shandong First Medical University (Shandong Eye Hospital), Jinan 250117, China

School of Ophthalmology, Shandong First Medical University & Shandong Academy of Medical Science, Jinan250117, China

E-mail: hgao@sdfmu.edu.cn

C. Wei and Y. Sun equally to this work.

Supporting Figures

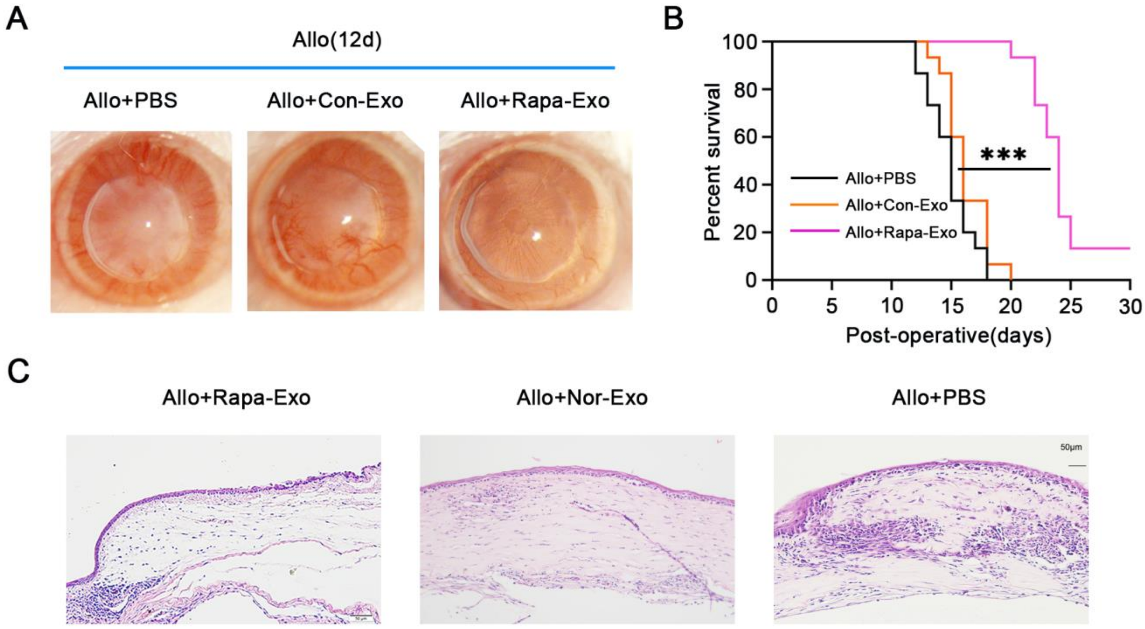

Figure S1. Comparison of anti-rejection effect between Nor-Exo and Rapa-Exo using corneal transplantation model. (A) The representative corneal grafts of different groups were observed using slit-lamp microscopy. (B) The survival of corneal grafts with different groups was analyzed by Kaplan-Meier method (n = 10/group). (C) The pathological alterations of corneal allografts under different treatments by H&E staining (n = 3/group). Scale bar 50 μm. Allo+PBS, the allogeneic group with subconjunctival treatment of PBS; Allo+Nor-Exo, the allogeneic group topically administrated with Nor-Exo (100 μg/mL); Allo+Rapa-Exo, the allogeneic group topically administrated with Rapa-Exo. **P<0.01.

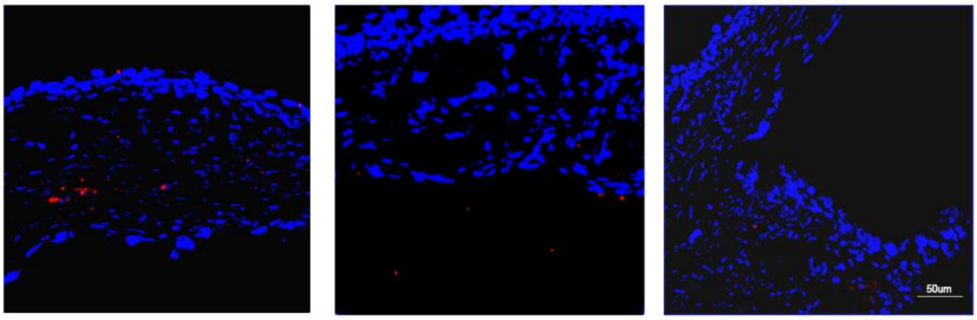

Figure S2. Distribution of Rapa-Exo in ocular tissues after subconjunctival injection. The distributions of EvLINK 555-labeled exosome after subconjunctival injection was evaluated using immunofluorescence staining. Scale bar 50 µm.

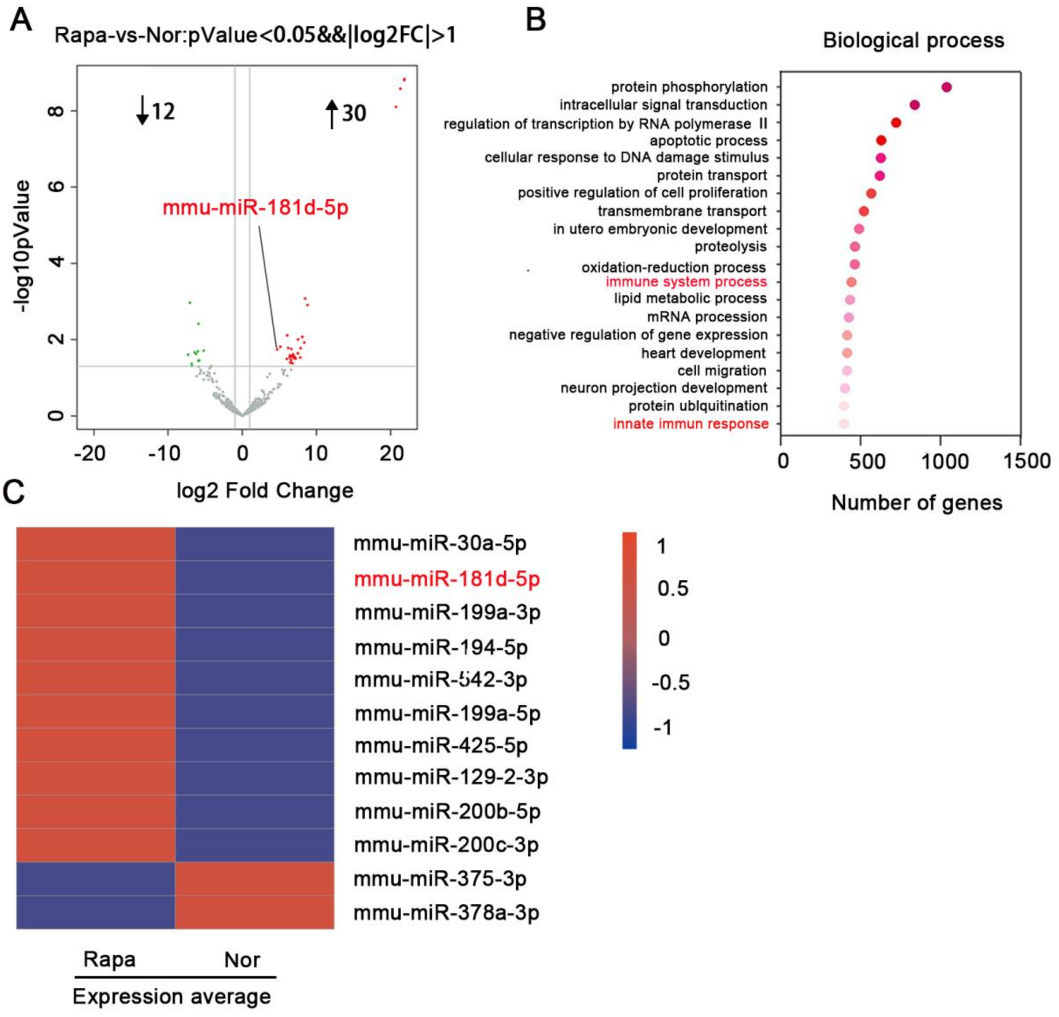

Figure S3. miRNA profile and characterization of Rapa-Exo. (A) Volcano plot showing the differentially-expressed miRNAs in Rapa-Exos. The red dots indicated the upregulated miRNAs, the blue dots denoted the down-regulated miRNAs. (B) The GO analysis of the predicted target genes of differentially-expressed miRNAs involving various biological processes (BPs). (C) The heatmap revealing the differentially-expressed miRNAs associated with innate immune responses and immune system process.

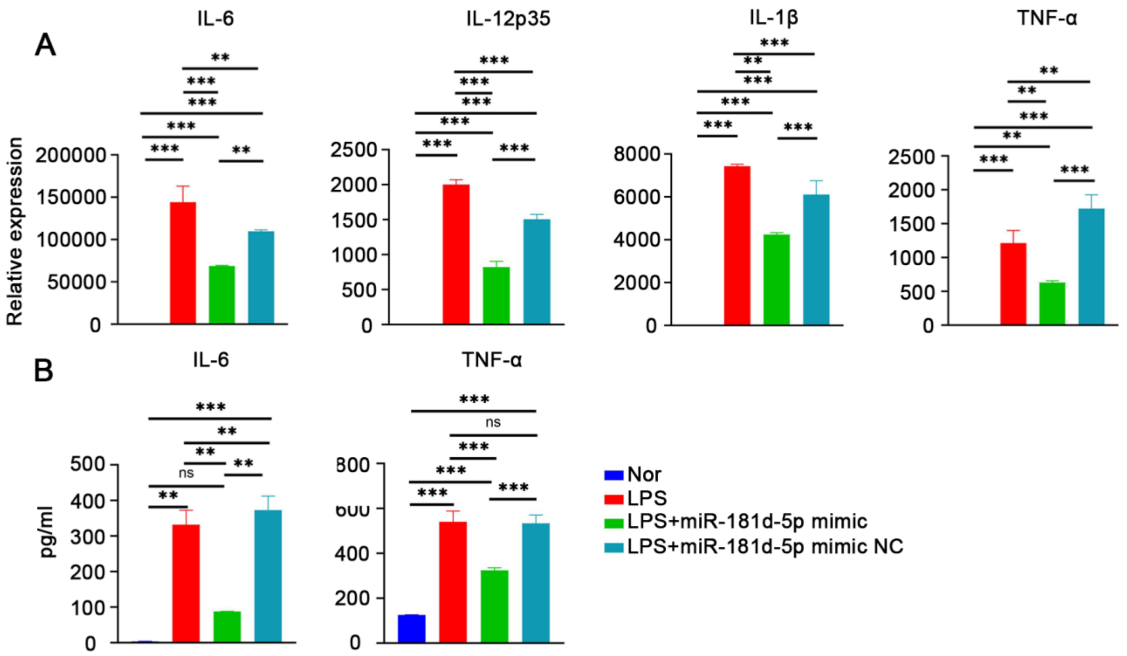

Figure S4. The effect of miR-181d-5p on LPS-induced inflammatory response in BMDMs. (A) The transcriptional levels of pro-inflammatory cytokines in LPS-challenged BMDMs with different treatments. (B) The protein levels of IL-6 and TNF-α in BMDM supernatant after treatment with different conditions. Nor, the untreated BMDMs; LPS, the BMDMs challenged with LPS (100 ng/mL); LPS+miR-181d-5p mimic, the BMDMs co-treated with LPS (100 ng/mL) and miR-181d-5p mimic (1000 μg/mL); LPS+miR-181d-5p mimic NC, the BMDMs stimulated with LPS (100 ng/mL) in presence of miR-181d-5p mimic NC(1000 μg/mL). ***P*<0.01, ****P*<0.001, n.s, no significance.

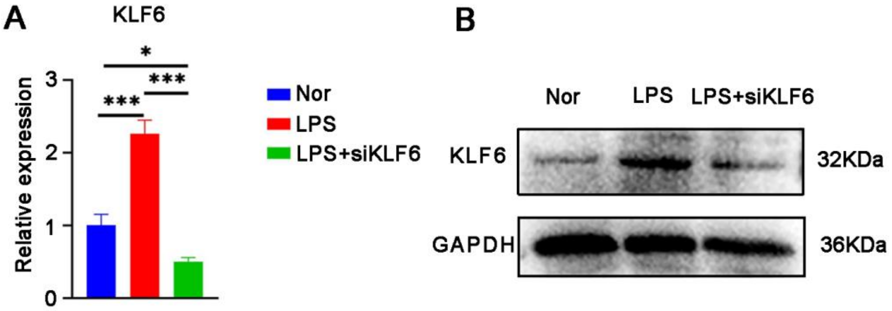

Figure S5. The knockdown of KLF6 using siRNA in BMDMs. (A) The transcriptional levels of KLF6 in LPS-challenged BMDMs after transfection of siKLF6. (B) WB analysis of KLF6 in LPS-stimulated BMDMs in the presence or absence of siKLF6. Nor, the untreated BMDMs; LPS, the BMDMs challenged with LPS (100 ng/mL); LPS+siKLF6, the BMDMs co-treated with LPS (100 ng/mL) and siKLF6 (1000 μg/mL). **P*<0.05, ****P*<0.001, n.s, no significance.

Supporting Table

Table S1. The sequences used for miR-181d-5p mimic, miR-181d-5p mimic NC, miR-181d-5p antagomir/inhibitor and siKLF6.

Name	Orientation	Sequences (5'→3')
miR-181d-5p mimic	sense	5'-UUUGUUCGUUCGGCUCGCGUGA-3'
miR-181d-5p mimic NC	sense	5'-UUCUCCGAACGUGUCACGUTT-3'
miR-181d-5p antagomir/inhibitor	sense	5'-ACCCACCGACAACAAUGAAUGUU-3'
siKLF6	sense	5'-GCUCCAGCUUUAAUUAUAATT-3'
	antisense	5'-UUAUAAUUAAAGCUGGAGCTT-3'

Table S2. The primers used for real-time PCR.

Name	Orientation	Sequences(5'→3')
GAPDH	Forward	5'-GGTGAAGGTCGGTGTGAACGGA-3'
	Reverse	5'-TGTTAGTGGGGTCTCGCTCCTG-3'
MU6	Forward	5'-CGCTTCGGCAGCACATATACTA-3'
	Reverse	5'-GGAACGCTTCACGAATTTGC-3'
miR-181d-5p	Forward	5'-GGCAACATTCATTGTTGTCGGT-3'
	Reverse	5'-GTCGTATCCAGTGCAGGGTCCGAGGTATTCGCACTGGATACGACacccac-3'
KLF6	Forward	5'-TTCCAACCCGACATGGATGT-3'
	Reverse	5'-CTCACTCTGAAGATAGCGTTCCAA-3'
IL-12p35	Forward	5'-GGGACCAAACCAGCACATTG-3'
	Reverse	5'-TACCAAGGCACAGGGTCATCA-3'
IL-1β	Forward	5'-GGGACCAAACCAGCACATTG-3'
	Reverse	5'-TGA TGT GCT GCT GGG AGA TT-3'
IL-17A	Forward	5'-GACTCTCCACCGCAATGAAGAC-3'
	Reverse	5'-CTCTTCAGGACCAGGATCTCTTG-3'
CCR7	Forward	5'-CTGGCCCTCTTCCTCTCCAT-3'
	Reverse	5'-GCACTAGGAACCCAAAAACCAT-3'
IL-6	Forward	5'-ACCACTCCCAACAGACCTGTCT-3'
	Reverse	5'-CAGATTGTTTTCTGCAAGTGCAT-3'
TNF-α	Forward	5'-ACAAGGCTGCCCCGACTAC-3'
	Reverse	5'-TGGGCTCATACCAGGGTTTG-3'
IFN-γ	Forward	5'-TGTTACTGCCACGGCACAGT-3'
	Reverse	5'-CTGGCTCTGCAGGATTTTCAT-3'
